# The evolutionary history of IGKC in mammals reveals ancient duplications and remarkable divergence in lagomorphs

**DOI:** 10.3389/fimmu.2025.1686094

**Published:** 2025-10-15

**Authors:** Ana Pinheiro, J. Ricardo Borges, João Pedro Marques, Pedro José Esteves

**Affiliations:** ^1^ CIBIO‐InBIO, Research Center in Biodiversity and Genetic Resources, University of Porto, Vairão, Portugal; ^2^ BIOPOLIS Program in Genomics, Biodiversity and Land Planning, Research Center in Biodiversity and genetic resources (CIBIO), Vairão, Portugal; ^3^ Department of Biology, Faculty of Sciences, University of Porto, Porto, Portugal; ^4^ CITS - Center of Investigation in Health Technologies, CESPU, Gandra, Portugal

**Keywords:** evolution, lagomorphs, *IGKC*, mammals, gene duplication

## Abstract

**Background:**

The Immunoglobulin Kappa Constant (*IGKC*) gene encodes the constant region of the immunoglobulin kappa light chain, a crucial component of antibodies. Despite its important biological role, the genetic information for this gene remains scarce, with data for only 16 mammal species (as of July 2025) fully characterized in the International ImMunoGeneTics information system (IMGT) database.

**Results:**

Using genomic sequences from NCBI and Ensembl, we expanded this to 124 *IGKC* sequences across 104 mammals, including two monotremes, eight marsupials, and 94 placentals. We uncovered unusual evolutionary dynamics in lagomorphs, showing independent *IGKC* duplications in *Ochotonidae* and *Leporidae*, giving rise to rabbit *IGKC1* and *IGKC2*. No conserved glycosylation sites were found, but 26 sequences from 14 species carried potentially N-glycosylated sites, including two new sites in lagomorphs. Selection analyses revealed pervasive purifying selection interspersed with codons under positive selection, while aBSREL identified episodic diversifying selection in several lineages.

**Conclusions:**

Our study represents a significant contribution to the knowledge of the IGKC gene, substantially expanding on information available in IMGT. It highlights complex evolutionary trajectories, especially in lagomorphs. The presence of N-glycosylated sites suggests potential effects on antigen binding, stability, or half-life. The coexistence of purifying and episodic positive selection points to a balance between structural conservation and lineage-specific functional diversification.

## 1 Introduction

Antibodies are typically Y-shaped glycoproteins composed of two distinct types of polypeptide chains, two identical heavy (H), and two identical light (L) chains. Each chain has a variable (V) and a constant (C) region. The light (VL and CL) chains and the variable (VH) and first constant domain of the heavy chains (CH1) constitute the antibody regions that recognizes and binds antigens (Fab regions), while the remaining constant domains of the heavy chains constitute the region that assures an effector function (Fc region). In mammals, there are two Ig L chain isotypes, kappa (K) and lambda (λ), which are functionally indistinguishable. Each L chain is encoded by three genes, variable (*VL*), joining (*JL*), and constant (*CL*), and each isotype has its own set of *VL*, *JL*, and *CL*. The constant region of the kappa light chain (*IGKC*) gene encodes the constant region of the immunoglobulin kappa light chain, a protein that interacts with the κ*VL*, and *JL* regions and contributes to the effector phase of humoral immunity by mediating the elimination of bound antigens.

Humans and mice have a single *IGKC*. In humans, the *IGKC* gene is located on chromosome 2 (2p11.2), while in mice it is located on chromosome 6 (6C1). In the European rabbit (*Oryctolagus cuniculus*), the *IGKC* and the joining region of the kappa light chain (*IGKJ*) have duplicated and originated two different kappa light chains (*IGKC1* and *IGKC2*) (1, 2). This duplication, confirmed by genomic data obtained from the OryCun 2.0 assembly, is located on chromosome 2 (3).

The genetic variation of the rabbit kappa light chains has been studied in detail and shows unique features. The *IGKC1* locus has an additional cysteine residue, C85.4 (International ImMunoGeneTics information system (IMGT) numbering), that links, through an extra disulfide bond, to *IGKV1* C96 (IMGT numbering) (4–6). Additionally, the European rabbit *IGKC1* has a unique glycosylation site, 85.1*NLS*86 (IMGT numbering). Glycosylation, the process of attaching sugar molecules (glycans) to proteins, plays a crucial role in the immune system. Glycosylation can affect protein folding, stability, and interactions with other molecules, including other proteins and receptors (7, 8). The degree of inter-allelic diversity of the rabbit *IGKC1* revealed high amino acid differences (9–12), only similar to that currently observed at vertebrate MHC loci (13). The trans-species nature, another benchmark of MHC evolution (14), was also documented for the rabbit b-locus allotypes obtained in *L. americanus* and European rabbit (15).

The information available for *IGKC* in the IMGT database remains limited, with data for only 16 mammal species (as of July 2025; https://www.imgt.org/IMGTrepertoire/Proteins/index.php#B). Recently, advances in genome sequencing technologies, particularly next-generation sequencing (NGS), have enabled the rapid sequencing and comparison of genomes across a wide range of species. This comparative genomics approach allows us to identify pathways that are unique to certain species, providing insights into their evolutionary history and adaptations. Evolutionary studies are crucial for understanding the mechanisms driving evolutionary innovation. In essence, the increased genetic information revealed by new genome sequencing technologies provides a powerful tool to understand the dynamics and creative processes of evolution. In this work, we sought to expand the knowledge about the *IGKC* genes in mammals (monotremes, marsupials, and placentals). Mining public databases, we retrieved *IGKC* sequences from over 100 mammal species and performed natural selection analyses. The results represent a major contribution to the study of this gene and provide an important increment to the IMGT database.

## 2 Materials and methods

Mammalian *IGKC* sequences were obtained from publicly accessible databases using our standard methodology, which we have already used in several studies (e.g (16–19). In total, we gathered 123 sequences from monotremes (two), marsupials (seven), and placentals (114). The accession numbers of all the sequences used are listed in **Supplementary Table S1**. The 114 placental sequences represent 94 species, with most species represented by a single sequence, except for lagomorphs, where several sequences per species were obtained (**Supplementary Table S1**). The sequences were retrieved through BLASTn searches in NCBI’s GenBank (http://www.ncbi.nlm.nih.gov/genbank/) and Ensembl (https://www.ensembl.org/index.html) genome databases, using as queries the mammalian *IGKC* sequences available in the IMGT database, which represent true *IGKC* with high confidence. For Ensembl-derived sequences, we used the *Homo sapiens IGKC* gene (ENSG00000211592) as a query and retrieved the list of mammalian orthologues available under the “Orthologues” section. As of July 2025, a total of 49 one-to-one orthologues and one one-to-two orthologue (in *Oryctolagus cuniculus*) were available. Because our BLASTn queries retrieved only annotated CDS, with transcript evidence, the initial two nucleotides of the IGKC exon (derived from the J–C splice junction) were not included in all sequences. As these do not alter the reading frame or translation, we expect no effect on functional inference, though small phylogenetic biases cannot be excluded. All obtained sequences contained the typical stop codon at the 3′ end; this was excluded from our analysis.

Sequences were aligned with Clustal W (20) as implemented in BioEdit (21), followed by visual inspection and necessary manual corrections. The final nucleotide sequence alignment is provided in **Supplementary Material: Data 1**. Amino acids were translated from the nucleotide sequences. Codon numbering is according to the IMGT unique numbering (22). N-linked glycosylation sites were estimated using the online tool NetNGlyc 1.0 Server, with + indicating a potential to reach the 0.5 threshold and ++/+++ to reach the 0.75 threshold (23).

### 2.1 Molecular phylogenetic analyses

A maximum likelihood (ML) phylogenetic tree was constructed for the mammalian nucleotide alignment using IQ-TREE v3.0.1. The best-fit substitution model was TVM+F+I+G4, selected under the Bayesian Information Criterion (BIC), which we preferred over AIC/AICc due to its stronger penalization of model complexity, thus reducing the risk of over-parameterization in large datasets. Node support was estimated using 10,000 ultrafast bootstrap replicates. A tree was inferred, and the tree topology was compared to the accepted mammalian phylogeny.

To further investigate the lagomorphs’ *IGKC* evolution, we constructed an ML phylogenetic tree for the lagomorph amino acid alignment using IQ-TREE v3.0.1. The best-fit substitution model was WAG+R4, again selected under BIC for its balance between model fit and parsimony. Node support was estimated using 10,000 ultrafast bootstrap replicates.

### 2.2 Detection of positive selection

Positive selection was assessed using HyPhy package v2.5.75. Codon-based analyses were performed on the nucleotide alignment comprising 108 codons. Four site-based methods were applied: SLAC (single-likelihood ancestor counting), FEL (fixed effects likelihood), FUBAR (fast, unconstrained Bayesian approximation), and MEME (mixed effects model of evolution). These methods allowed the detection of pervasive and episodic selection acting across codon sites. Additionally, the aBSREL (adaptive branch-site random effects likelihood) model was used to detect episodic diversifying selection on individual branches of the phylogenetic tree.

Sites under pervasive positive and purifying selection were mapped onto the three-dimensional structure of the human IGKC protein, predicted by AlphaFold (UniProt entry P01834), using the high-confidence regions of the model for visualization.

## 3 Results

**Figure 1:**
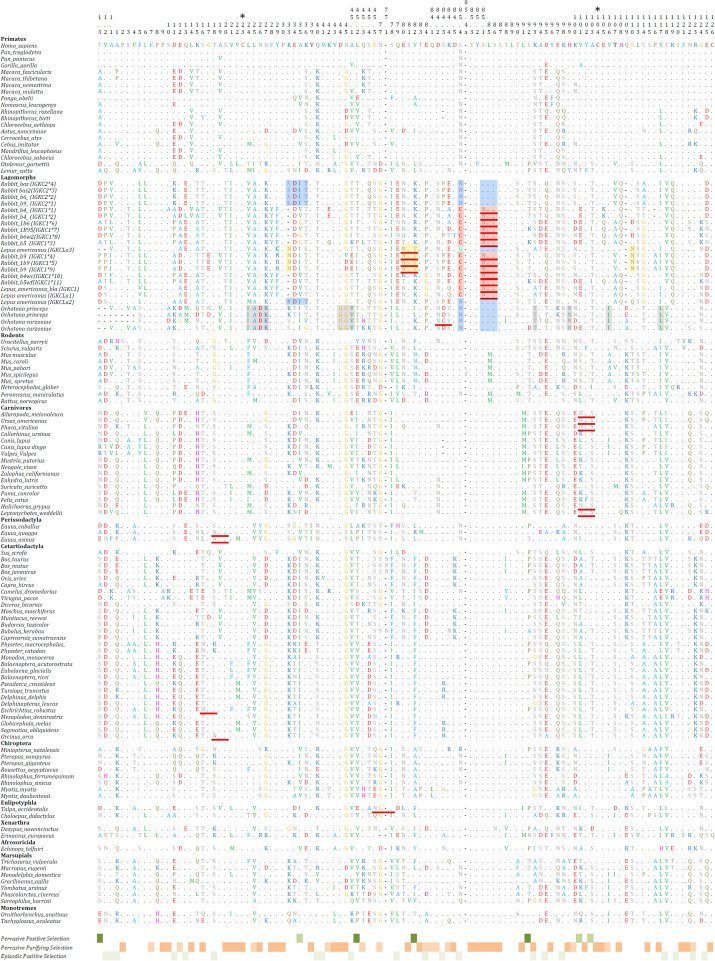
Mammalian *IGKC* amino acid sequences alignment. The full *IGKC* amino acid sequences are depicted. The codon numbering is according to the IMGT unique numbering for the constant domain (22). Asterisks (*) above the numbering indicate the position of conserved Cys residues. For Lagomorphs, characteristic residues for *IGKC1* are shaded in red, *IGKC1* b9 alleles are shaded in yellow, *IGKC2* are shaded in blue, *IGKCLa3* are shaded in light blue, and Ochotonidae *IGKC* are shaded in gray. N-glycosylation sites identified using the online tool NetNGlyc 1.0 (23) are underlined in red. Dots (.) represent identity with the top sequence, and dashes (–) indicate gaps in the alignment. The codons identified as under pervasive positive selection, pervasive negative selection and episodic positive selection are shown under the alignment.

**Figure 2:**
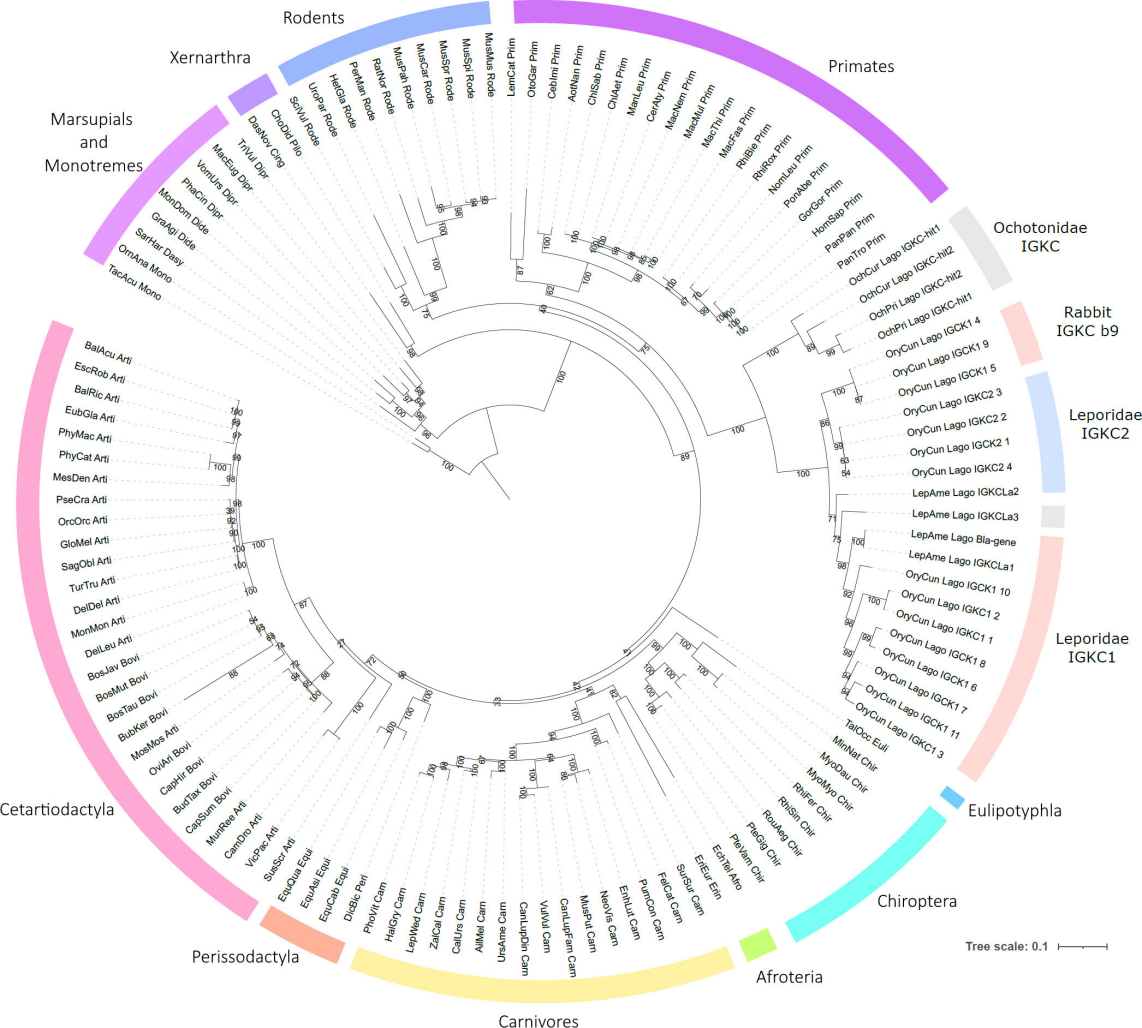
Phylogenetic tree of mammalian *IGKC*. The nucleotide phylogenetic tree was obtained using TVM+F+I+G4 as the best-fit substitution model, selected under the Bayesian Information Criterion (BIC). Node support was estimated using 10,000 ultrafast bootstrap replicates. All bootstrap values are shown, with values indicated near the most relevant branches. *IGKC1* is shaded in pink, *IGKC2* is shaded in blue, and indeterminate lagomorphs’ *IGKC* is shaded in gray.

The obtained 123 *IGKC* sequences represent 105 mammalian species (95 placentals, seven marsupials, and two monotremes) (**Figure 1**). For the vast majority of mammalian species analyzed, we found only a single copy of this gene. The exception was lagomorphs, in which at least two sequences were recovered for each species. In our alignment, to the four *IGKC2* and nine *IGKC1* European rabbit alleles described in IMGT, we added two more previously published alleles, b4wc and b5wf (11), here named as rabbit IMGT IGKC1*10 and IMGT IGKC1*11, respectively. For *Ochotona* species, we obtained four novel sequences: two for *O. princeps* and two for *O. curzoniae*. Regarding *Lepus americanus*, we identified three new sequences in addition to the previously described *L. americanus IGKC* sequence (15). All sequences that we obtained contain a stop codon at the 3′ end, have high homology among them and the queries, and, as annotated CDS, have derived transcripts. Additionally, the diversity of genes that we found agrees with what is known about the IGKC; mammalian species have one IGKC except for the rabbit, which has two IGKC. The *IGKC* nucleotide ML tree generally conforms to the accepted mammalian phylogeny (24) (**Figure 2**). Within lagomorphs, the *Ochotona* sequences occupy a basal position (100 bootstrap support), while the rabbit and *Lepus* sequences form a second cluster (100 bootstrap support). Within this cluster, three groups are evident: (a) the rabbit *IGKC2* cluster (99 bootstrap support), (b) the rabbit *IGKC1* b9 alleles (IGKC1*4, IGKC1*5, and IGKC1*9) (100 bootstrap support), and (c) the remaining rabbit *IGKC1* and the *Lepus* sequences (71 bootstrap support) (**Figure 2**).

### 3.1 The special case: lagomorphs

**Figure 3:**
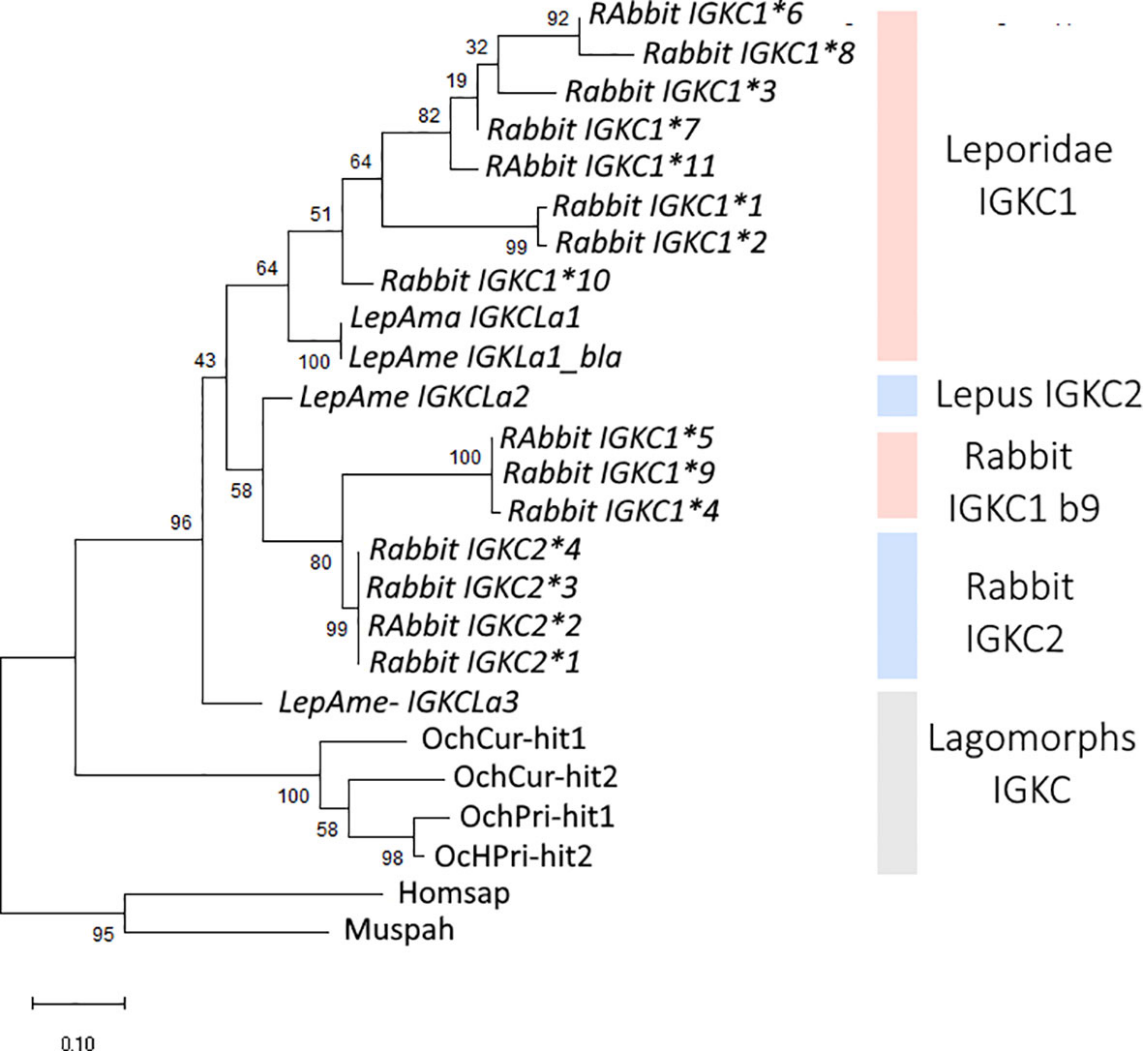
Phylogenetic tree of lagomorphs’ *IGKC*. The amino acid phylogenetic tree was obtained using WAG+R4 as the best-fit substitution model, selected under the Bayesian Information Criterion (BIC). Node support was estimated using 10,000 ultrafast bootstrap replicates. All bootstrap values are shown, with values indicated near the most relevant branches. *IGKC1* is shaded in pink, *IGKC2* is shaded in blue, and indeterminate lagomorphs’ *IGKC* is shaded in gray. The scale bar refers to the inferred amount of change per site along branches. Rabbit IGKC alleles are indicated with *n.

We obtained *IGKC* genes for four lagomorph species: *Ochotona prínceps*, *Ochotona curzoniae*, *Lepus americanus*, and *Oryctolagus cuniculus.* The lagomorphs are divided into two families, *Leporidae* and *Ochotonidae*. *Ochotonidae* has a single genus, *Ochotona* (pikas), while *Leporidae* includes 10 genera, among them *Lepus* and *Oryctolagus* (25). The lagomorph *IGKC* amino acid tree shows a different pattern than the nucleotide phylogeny (**Figures 2**, **3**, respectively). In the amino acid tree (**Figure 3**), the *Ochotona* sequences again occupy a basal position (100 bootstrap support), with rabbit and *Lepus* sequences forming a second cluster (96 bootstrap support). Within Leporidae, the *L. americanus* sequence *IGKCLa3* occupies a basal position. Some *IGKC1* alleles, the b9 alleles—IGKC1*4, IGKC1*5, and IGKC1*9—form a well-supported cluster (100 bootstrap support) grouping with *IGKC2* (99 bootstrap support), apart from other *IGKC1* alleles (80 bootstrap support). Previous phylogenetic analyses have also placed the *IGCK1 b9* alleles closer to *IGKC2* than to other *IGKC1* (13). Although with a lower bootstrap support, *L. americanus IGKCLa2* clusters with the *IGKC2* and *IGKC1* b9 alleles, while *IGKCLa1* clusters with *IGKC1* (58 and 64 bootstrap support, respectively).

**Figure 4:**
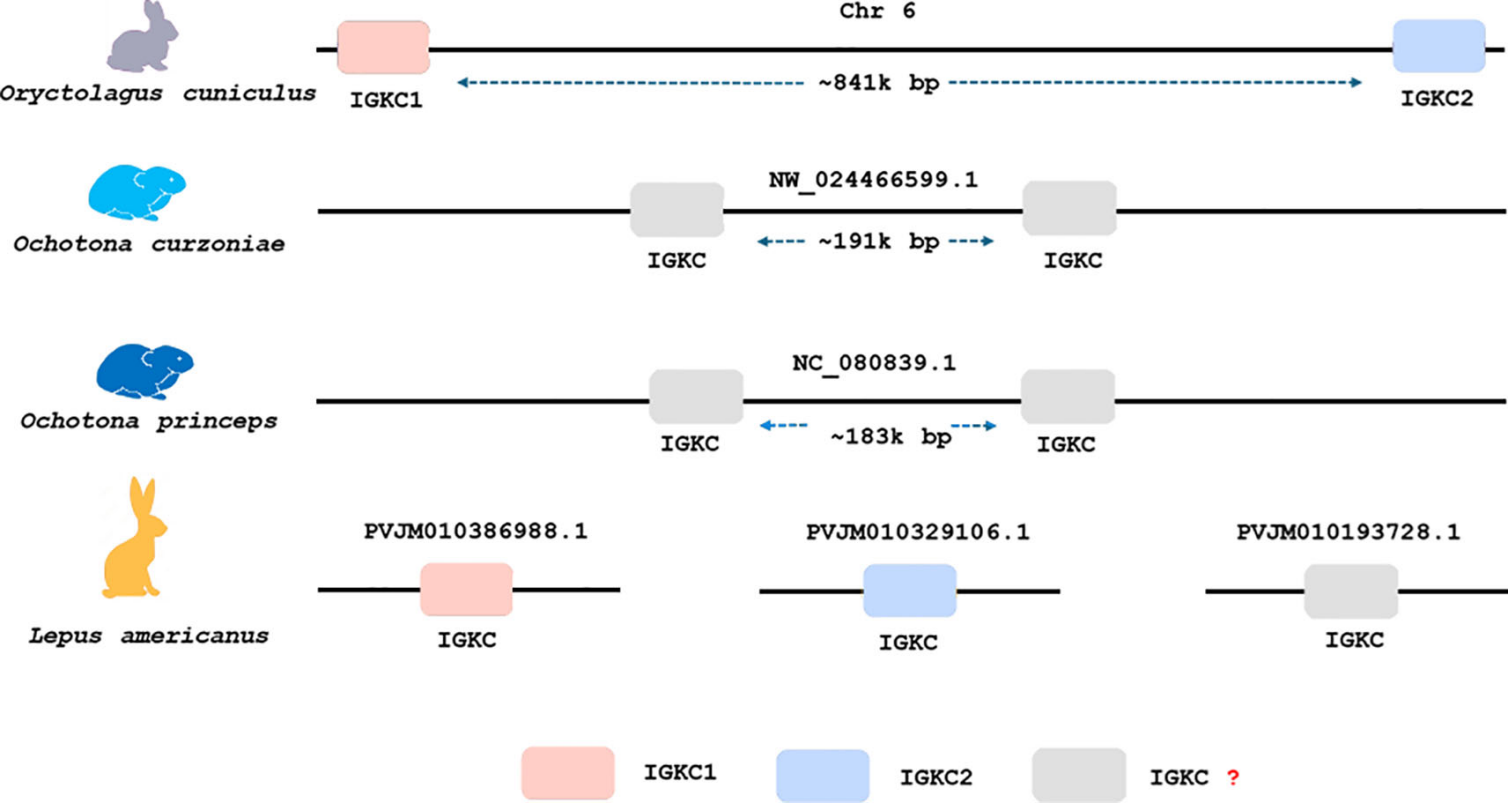
Genomic organization and copy number of *IGKC* genes in four lagomorph species. Each schematic represents one species: *Oryctolagus cuniculus* (European rabbit), *Ochotona curzoniae* (plateau pika), *Ochotona princeps* (American pika), and *Lepus americanus* (snowshoe hare), from top to bottom. The boxes indicate *IGKC* gene copies and their relative chromosomal positions based on BLASTn results.

In both Ochotona species, we found two *IGKC* genes that were located chromosomally adjacent (**Figure 4**), similarly to the arrangement described for the European rabbit (3). The Ochotona *IGKC* sequences have characteristic residues distinguishing them from Leporidae *IGKC*: 24*IADK*27, 44*GGV*45.1, *N*99, *I*106, and *L*117 (**Figure 1**). These sequences also share rabbit *IGKC2* residues *N*85.4 and 85.1*SLS*86 (**Figure 1**), supporting the view that *IGKC2* predates *IGKC1* (13).

For *Lepus americanus*, we identified three new sequences (**Figure 1**). As the genome assembly for this species is not a chromosome-level assembly, the three IGKC sequences are located on separate scaffolds, preventing us from determining whether they represent three gene copies or two genes with one allelic variant. The nucleotide and amino acid phylogenies (**Figures 2** and **3**, respectively) show two different scenarios for the leporid *IGKC*. In the nucleotide tree, all *L. americanus* sequences cluster with rabbit *IGKC1* sequences (**Figure 1**), whereas in the amino acid tree *L. americanus IGKCLa1* clusters with rabbit *IGKC1* sequences and *IGKCLa2* clusters with the rabbit *IGKC2* sequences. The *Lepus americanus IGKCLa1* shares two distinguishing features of the European rabbit *IGKC1:* C85.4 and 85.1*NLS*86. This shows that the rabbit *IGKC1* novelty features, C85.4 and 85.1*NLS*86, that may have triggered the high allelic diversity observed in European rabbit *IGKC1* (reviewed in (13)) were already present in *L. americanus* and emerged at least 12 million years ago (*Oryctolagus*–*Lepus* divergence time (26, 27);). The second *L. americanus* sequence, named here *L. americanus IGKCLa2*, surprisingly shares with the European rabbit *IGKC2* residues *N*85.4 and 85.1*SLS*86 as well as 33*SDIT*36. The *L. americanus IGKCLa3* shares some characteristic residues with the rabbit b9 alleles, IGKC1*4, IGKC1*5, and IGKC1*9, such as *N*33, 80*NST*82, and *N*110, but lacks the *IGKC1* 85.1*NLS*86 motif, having instead 85.1*SLS*86 like rabbit *IGKC2* and unique features *C*29 and *G*85.4.

### 3.2 *IGKC* glycosylation sites

Glycosylation plays a major role in immunoglobulin structure and function. Heavy chain isotypes, IgA, IgG, IgE, IgD, and IgM, are known to be N-glycosylated to various extents (reviewed in (28), but *IGKC* glycosylation has not been described previously. We screened the mammalian *IGKC* sequences for N-linked glycosylation sites. No conserved sites of glycosylation were found for mammalian *IGKC*; however, 26 sequences, representing 14 species, are potentially N-glycosylated (**Figure 1**). *Equus quagga* and *Equus asinus* (Perissodactyla) (NetNGlyc threshold: ++), *Orcinus orca* (Cetartiodactyla) (NetNGlyc threshold: ++), and *Miniopterus natalensis* (Chiroptera) (NetNGlyc threshold: +) share the 18*NAS*20 glycosylation site, and five Carnivores have 101*NFS*103 (NetNGlyc threshold: +). *Eschrichtius robustus IGKC* has *16NGT18* (NetNGlyc threshold: +++) and *Talpa occidentalis* has 45.5*NGS*78 (NetNGlyc threshold: +).

In lagomorphs’ *IGKC*, a potential N-glycosylation site has been identified as a hallmark characteristic of *IGKC1*, 85.1*NLS*86 (15) (NetNGlyc threshold: ++). Our analysis reveals additional putative N-glycosylation sites. The rabbit b9 alleles, IGKC1*4, IGKC1*5, and IGKC1*9, are potentially glycosylated at 80*NST*82 and 85.1*NLS*86 (NetNGlyc threshold: ++). *L. americanus IGKCLa1* is also potentially glycosylated at 85.1*NLS*86 (NetNGlyc threshold: +), while *IGKCLa3* can be glycosylated at 80*NST*82 (NetNGlyc threshold: ++). All *Ochotona* sequences have a 84.2*NXS/T*84.4 motif, but our results indicate that only one *O. curzoniae* sequence is potentially glycosylated at this position, having the motif 84.2*NCSD*84.4 (NetNGlyc threshold: +).

### 3.3 Positive selection analyses

The analysis of selection pressures acting on *IGKC* coding sequences identified multiple codon sites under pervasive and episodic positive selection. Seven codons (1.3, 35, 45.2, 82, 92, 101, and 103) were supported by at least two site-based methods (SLAC, FEL, and FUBAR), while MEME revealed episodic selection in 25 codons, including those previously identified. Codon 85.4, which corresponds to one of the key motifs distinguishing rabbit IGKC1, was flagged as under selection.

Widespread purifying selection was also detected, with 53 codons identified across SLAC and FEL as being subject to significant evolutionary constraint. These included codons located in highly conserved regions of the constant domain, such as residues 2, 21–23, 28–30, 81, 84.5, 85.3–87, 91, 93, 96, 99, 102, 104–106, and 115–126. These positions likely reflect structural or functional constraints essential to the immunoglobulin fold.

**Figure 5:**
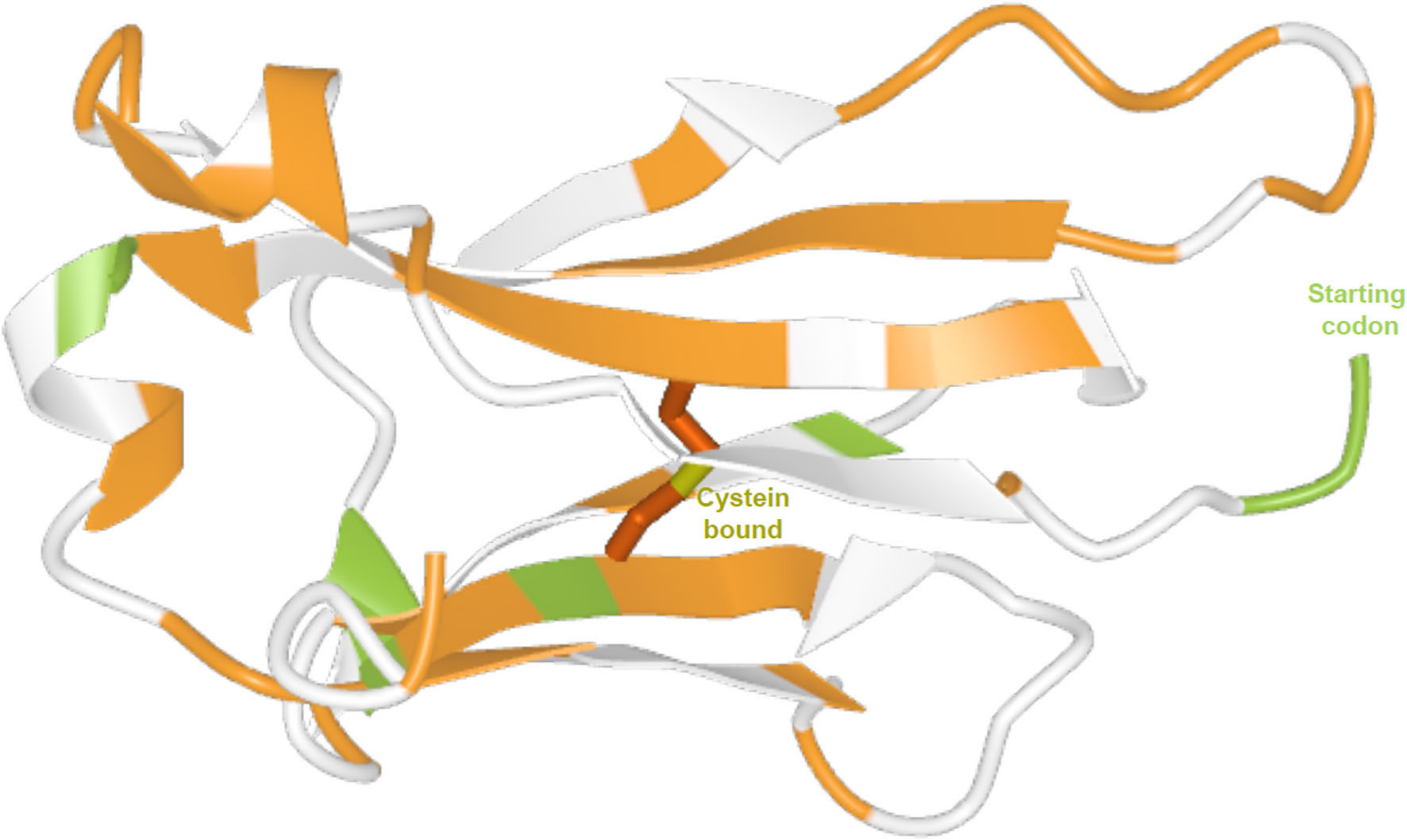
Three-dimensional structure of the human IGKC protein. Predicted by AlphaFold (UniProt entry P01834, model with very high confidence). Sites under pervasive positive selection are shown in green, and sites under purifying selection are shown in orange.

The sites under pervasive positive and purifying selection are distributed across the protein. The sites identified as under pervasive positive selection are generally located in exposed regions, in the loops or loop vicinity (**Figure 5**).

Branch-site analyses using aBSREL detected episodic diversifying selection in the terminal branches of *Sarcophilus harrisii* and *Equus asinus* as well as in several internal nodes: between *Vulpes vulpes* and *Canis lupus dingo*, between one copy of *Ochotona curzoniae* and the two *O. princeps* sequences, and in two clades within the rabbit IGKC1 group. These results support the notion that selective pressures are not uniformly distributed across mammalian lineages.

## 4 Discussion

Antibodies are hallmark molecules of the vertebrate immune system, playing a crucial role in protective immunity. The heavy chains, which determine the antibody isotypes, have been widely studied, with their genetic diversity, structure, function, and evolution well characterized (reviewed in, e.g (29–31)) and continue being studied as new evidences of clinical relevance continue emerging. On the contrary, the two light chains, kappa (K) and lambda (λ), have been comparatively neglected, and much less is known about their diversity and evolution. In this work, we sought to extend the knowledge on the evolution of mammalian *IGKC* by mining available genome assemblies and conducting natural selection analyses.

We recovered sequences for 104 mammalian species, enhancing our understanding of *IGKC* genetic diversity. We acknowledge that the accuracy of available genome assemblies is a limiting factor. Assembly errors, particularly in fragmented genomes such as *Lepus americanus*, may underlie some ambiguities in copy number or motif conservation. Nevertheless, synteny, motif analysis, and phylogenetic consistency strongly support our identifications. Our data revealed the duplication of this gene in lagomorphs and confirmed that such duplication is unique to this group. The *IGKC* was previously shown to be duplicated in the European rabbit, which carries two *IGKC* copies, *IGKC1* and *IGKC2* (1–3). The presence of two *IGKC* genes in *Ochotona* and *Oryctolagus* could be explained by a duplication in the lagomorph ancestor between 50 to 57.2 million years ago (26, 32, 33). In that scenario, one *Ochotona* gene would be expected to cluster with rabbit *IGKC1* and the other with rabbit *IGKC2*. However, both the nucleotide and amino acid sequence phylogenies (**Figures 2** and **3**) place the *Ochotona* sequences together in a basal position relative to all lagomorph *IGKC*. This pattern suggests that the *Ochotona IGKC* duplication likely occurred in the *Ochotona* ancestor, after the *Leporidae* and *Ochotonidae* split. A second *IGKC* duplication would then have taken place in the *Leporidae* ancestor, leading to the emergence of rabbit *IGKC1* and *IGKC2.* Despite the incongruences between the lagomorph *IGKC* nucleotide and amino acid trees, we consider the most likely scenario to be a duplication in the *Leporidae* ancestor, as supported by the amino acid data and shared diagnostic residues between *Lepus* and *Oryctolagus* sequences.

In the nucleotide phylogeny, all three *L. americanus IGKC* sequences cluster with the rabbit *IGKC1*, except for IGKC1*4, IGKC1*5, and IGKC1*9 (**Figure 1**). In the amino acid tree, however, only the *L. americanus IGKCLa1* maintains this position; *IGKCLa1*, but not *IGKCLa2 and IGKCLa3*, shares the two distinguishing features of the European rabbit *IGKC1*: C85.4 and 85.1*NLS*86. This indicates that these rabbit *IGKC1* novelty features, 85.4*C* and 85.1*NLS*86, which may have triggered the high allelic diversity observed in European rabbit *IGKC1* (reviewed in (13)), were already present in the *Leporidae* ancestor 12 million years ago (*Oryctolagus–Lepus* divergence time (26, 27)). The *IGKCLa1* gene is identical to the *L. americanus bla* allele (**Figure 1**) described by Bouton and van der Loo (15). The *bla* allele is more closely related to the European rabbit *b4wc* allele (IGKC1*10) than the *b4wc* allele is to other European rabbit *IGKC1* alleles (15). This pattern is an example of a trans-species polymorphism. Together with other examples also described between the European rabbit and *Lepus* species in *IGHV* (34, 35) and *IGHA* (36), these are some of the rare instances of trans-species polymorphisms described outside of MHC genes (14, 37). In the amino acid tree, *L. americanus IGKCLa2* instead clusters with rabbit *IGKC2*. *IGKCLa2* shares residues *N*85.4 and 85.1*SLS*86, as well as 33*SDIT*36, with the European rabbit *IGKC2*. The *L. americanus IGKCLa3* shares some characteristic residues with the rabbit b9 alleles, IGKC1*4, IGKC1*5, and IGKC1*9, but lacks the *IGKC1* 85.1*NLS*86 motif, having instead 85.1*SLS*86 like rabbit *IGKC2*; it also has unique residues. The sharing of residues with *IGKC1* and *IGKC2* and its singularities cause it to adopt a basal position to other leporid *IGKC.*

Taken together, the most parsimonious explanation is that the second duplication in lagomorphs’ *IGKC* happened in the *Leporidae* ancestor, as reflected in the amino acid phylogeny and shared diagnostic residues between rabbit and *Lepus* sequences. The clustering of the *Lepus IGKC* with rabbit *IGKC1* in the nucleotide tree is likely the result of high homology observed in several regions of the *Lepus IGKC* sequences, which are probably being homogenized through concerted evolution (see **Supplementary Material: Data** 1). Concerted evolution can explain incongruences between nucleotide and amino acid phylogenetic trees when gene conversion and unequal crossing-over events homogenize sequences within a multigene family, leading to a pattern where paralogous genes within a species are more similar to each other than to their orthologous counterparts in different species (38, 39).

Glycans are found attached to the Fc tail of all Ig isotypes comprising 2%–14% of the Ig molecular weight depending on the Ig isotype (40). The Fab arms may also carry N-glycans; IgA, IgE, and IgM heavy chains are glycosylated in the CH1 domain (40), and additionally, 15%–25% of circulating IgG are glycosylated on the V regions (VL or VH) (41). The glycosylation sites in the V regions are only acquired during antibody maturation through somatic hypermutation, and research indicates that selection pressure favors the acquisition of Fab N-glycosylation sites during B cell affinity maturation (28). The light chain C regions’ glycosylation has not, to our knowledge, been investigated. Our analysis reveals that putative N-glycosylation sites are present in ~21% of *IGKC* (26 out of 123 IGKC sequences carry N-glycosylation sites). N-linked glycans in the Fab region are known to influence antigen binding, increase antibody stability, or extend the antibody half-life (28, 42, 43). All identified sequences with N-glycosylation sites carry only one putative site, except for the European rabbit *IGKC1* b9 alleles, IGKC1*4, IGKC1*5, and IGKC1*9, which carry two putative N-glycosylation sites, at 80*NST*82 and 85.1*NLS*86. The b9 alleles—IGKC1*4, IGKC1*5, and IGKC1*9—have been described to form an alternative bond between 84.5C and *IGKJ* in contrast to other *IGKC1* alleles that form a bond between 84.5C and *IGKV.* A possible explanation for this alternative bond would be the presence of a glycan at 80*NST*82. The sharing of glycosylation sites between *Lepus* and rabbit further supports the common origin of *IGKCLa1* and rabbit *IGKC1*. Intriguingly, the higher prevalence of IgG Fab glycans has been associated with the development of several autoimmune chronic diseases, namely, rheumatoid arthritis (RA), systemic lupus erythematosus, myasthenia gravis, pemphigus vulgaris, and ANCA-associated vasculitis (44, 45). The role of Fab glycans in autoimmunity is still unclear. On one hand, it has been shown that Fab glycans reduce the binding affinity for autoantigens (46, 47). Conversely, Fab glycans were found to enhance B cell receptor signaling and maintain its surface expression longer after antigenic stimulation (47). A shift toward the pro-inflammatory IgA2 subclass is also observed in RA patients with increased disease activity. IgA2 has twice as many N-glycosylation sites as IgA1, including a CH1 N-glycosylation site, and differences in their glycosylation pattern exist (48). Whether the glycan attached to the IgA2 CH1 N-glycosylation site has a direct influence on the pro-inflammatory nature of IgA2 has not been investigated.

### 4.1 Signatures of positive selection and functional relevance

The presence of codons under positive selection across diverse mammalian lineages indicates that IGKC, while structurally conserved, is subject to functional diversification. Several of the positively selected sites identified here lie within or adjacent to known functional motifs, including codon 85, which forms part of the unique glycosylation motif 85.1NLS86 in rabbit IGKC1. Its recurrent detection across SLAC, FEL, FUBAR, and MEME strongly suggests adaptive relevance. Similarly, branch-site tests identified episodic positive selection in specific taxa, including *Sarcophilus harrisii*, *Equus asinus*, and members of *Ochotona* and *Oryctolagus*, highlighting lineage-specific pressures potentially linked to immune challenges or structural innovation. In *S. harrisii*, this may reflect unique selective pressures associated with transmissible cancers, which have profoundly shaped the immune gene evolution in this species (49). In *E. asinus*, a domesticated species with a long history of close contact with humans and livestock, episodic selection may relate to exposure to diverse pathogen communities or to immune modulation under domestication (50).

In contrast, the strong signal of purifying selection across more than 50 codon sites points to a substantial functional constraint within much of the IGKC coding region. These conserved positions likely correspond to residues essential for maintaining the structural integrity of the kappa constant domain, particularly within β-strand regions and core elements of the immunoglobulin fold. The coexistence of strong purifying selection and episodic positive selection suggests a balance between preserving structural stability and allowing for lineage-specific functional adaptations, especially in clades where gene duplications or increased allelic diversity are observed.

## 5 Conclusion

This work substantially increases the information available for the *IGKC* gene and makes an important contribution to updating the IMGT database. Our findings show that lagomorphs present a unique evolutionary pattern for the *IGKC* gene, distinct from that observed in other mammals. However, the evolutionary history of *IGKC* genes within leporids could be even more complex. Therefore, obtaining IGKC sequences for additional leporids will be vital to understand the full evolutionary history of this gene in lagomorphs. Though there are no conserved N-glycosylation sites in *IGKC*, we detected several N-glycosylation sites in different mammalian species, some shared between species, which suggests that these glycosylation sites may have an important biological role. The presence of codons under positive selection across diverse mammalian lineages indicates that *IGKC*, while structurally conserved, is subject to functional diversification. Furthermore, the detection of episodic positive selection in specific taxa highlights lineage-specific pressures potentially linked to immune challenges or structural innovation.

## Data Availability

The original contributions presented in the study are included in the article/**Supplementary Material**. Further inquiries can be directed to the corresponding author.
