# Supplementary material for: The evolutionary history of IGKC in mammals reveals ancient duplications and remarkable divergence in lagomorphs
Source: Front Immunol. 2025 Oct 15;16:1686094. doi: 10.3389/fimmu.2025.1686094 (PMC12568662; doi:10.3389/fimmu.2025.1686094)
Supplement: Supplementary file 1 [file DataSheet1.zip › Supplementary Material_IGKC.docx]

Supplementary Material

# Supplementary Data

**Supplementary Data 1.** Alignment of the mammals' *IGKC* nucleotide sequences used in this study.

# Supplementary Tables

**Supplementary Table S1.** Accession numbers for the mammalian IGKC sequences obtained in the NCBI and ENSEMBL databases.

| **Common name** | **Scientific name** | **Accession number** | **Source** | **Acronym** |  |  |  |
| --- | --- | --- | --- | --- | --- | --- | --- |
| **Primates** | | | |  |  |  |  |
| Human | *Homo sapiens* | AH002839 | NCBI | Homsap |  |  |  |
| Chimpanzee | *Pan troglodytes* | ENSPTRG00000047067 | Ensembl | Pantro |  |  |  |
| Bonobo | *Pan paniscus* | ENSPPAG00000008361 | Ensembl | Panpan |  |  |  |
| Gorilla | *Gorilla gorilla* | BK063601 | NCBI | Gorgor |  |  |  |
| Crab-eating macaque | *Macaca fascicularis* | XM_065527520 | NCBI | Masfas |  |  |  |
| Tibetan macaque | *Macaca thibetana* | XM_050755255 | NCBI | Macthi |  |  |  |
| Pig-tailed macaque | *Macaca nemestrina* | ENSMNEG00000037667 | Ensembl | Macnem |  |  |  |
| Rhesus macaque | *Macaca mulatta* | BK063716 | NCBI | Macmul |  |  |  |
| Sumatran orangutan | *Pongo abelii* | BK063599 | NCBI | Ponabe |  |  |  |
| Gibbon | *Nomascus leucogenys* | ENSNLEG00000030584 | Ensembl | Nomleu |  |  |  |
| Golden snub-nosed monkey | *Rhinopithecus roxellana* | ENSRROG00000036801 | Ensembl | Rhirox |  |  |  |
| Black snub-nosed monkey | *Rhinopithecus bieti* | ENSRBIG00000030948 | Ensembl | Rhibie |  |  |  |
| Grivet | *Chlorocebus aethiops* | OQ624941 | NCBI | Chlaet |  |  |  |
| Ma's night monkey | *Aotus nancymaae* | ENSANAG00000019679 | Ensembl | Aotnan |  |  |  |
| Sooty mangabey | *Cercocebus atys* | ENSCATG00000042749 | Ensembl | Ceraty |  |  |  |
| Panamanian white-faced capuchin | *Cebus imitator* | ENSCCAG00000003458 | Ensembl | Cebimi |  |  |  |
| Drill | *Mandrillus leucophaeus* | ENSMLEG00000022333 | Ensembl | Manleu |  |  |  |
| Vervet-AGM | *Chlorocebus sabaeus* | ENSCSAG00000016459 | Ensembl | Chlsab |  |  |  |
| Bushbaby | *Otolemur garnettii* | ENSOGAG00000024766 | Ensembl | Otogar |  |  |  |
| Ring-tailed lemur | *Lemur catta* | BK063598 | NCBI | Lemcat |  |  |  |
| **Lagomorphs** | | | |  |  |  |  |
| European rabbit IGKC2*4 | *Oryctolagus cuniculus* | V00885 | NCBI | Orycun |  |  |  |
| European rabbit IGKC2*3 | *Oryctolagus cuniculus* | X56061 | NCBI | Orycun |  |  |  |
| European rabbit IGKC2*2 | *Oryctolagus cuniculus* | X05800 | NCBI | Orycun |  |  |  |
| European rabbit IGKC2*1 | *Oryctolagus cuniculus* | X05801 | NCBI | Orycun |  |  |  |
| European rabbit IGKC1*1 | *Oryctolagus cuniculus* | K01360 | NCBI | Orycun |  |  |  |
| European rabbit IGKC1*2 | *Oryctolagus cuniculus* | AH001227 | NCBI | Orycun |  |  |  |
| European rabbit IGKC1*3 | *Oryctolagus cuniculus* | K01363 | NCBI | Orycun |  |  |  |
| European rabbit IGKC1*4 | *Oryctolagus cuniculus* | X00674 | NCBI | Orycun |  |  |  |
| European rabbit IGKC1*5 | *Oryctolagus cuniculus* | X03050 | NCBI | Orycun |  |  |  |
| European rabbit IGKC1*6 | *Oryctolagus cuniculus* | M37809 | NCBI | Orycun |  |  |  |
| European rabbit IGKC1*7 | *Oryctolagus cuniculus* | M22542 | NCBI | Orycun |  |  |  |
| European rabbit IGKC1*8 | *Oryctolagus cuniculus* | Z48308 | NCBI | Orycun |  |  |  |
| European rabbit IGKC1*9 | *Oryctolagus cuniculus* | K01359 | NCBI | Orycun |  |  |  |
| European rabbit IGKC1*10 | *Oryctolagus cuniculus* | AJ222663 | NCBI | Orycun |  |  |  |
| European rabbit IGKC1*11 | *Oryctolagus cuniculus* | AJ222664 | NCBI | Orycun |  |  |  |
| Snowshoe hare bas allele | *Lepus americanus* | Z80231 | NCBI | Lepame |  |  |  |
| Snowshoe hare | *Lepus americanus* | PVJM010329106 | NCBI | Lepame |  |  |  |
| Snowshoe hare | *Lepus americanus* | PVJM010193728 | NCBI | Lepame |  |  |  |
| Snowshoe hare | *Lepus americanus* | PVJM010386988 | NCBI | Lepame |  |  |  |
| American pika | Ochotona princeps | NC_080839 | NCBI | Ochpri |  |  |  |
| American pika | Ochotona princeps | NC_080839 | NCBI | Ochpri |  |  |  |
| Plateau pika | Ochotona curzonae | NW_024466599 | NCBI | Ochcur |  |  |  |
| Plateau pika | Ochotona curzonae | NW_024466599 | NCBI | Ochcur |  |  |  |
| **Rodents** | | | |  |  |  |  |
| Arctic ground squirrel | *Urocitellus_parryii* | ENSUPAG00010018047 | ENSEMBL | Uropar |  |  |  |
| Eurasian red squirrel | *Sciurus_vulgaris* | ENSSVLG00005019486 | ENSEMBL | Scivul |  |  |  |
| House mouse | *Mus musculus* | V00807 | NCBI | Musmus |  |  |  |
| Ryukyu mouse | *Mus_caroli* | MGP_CAROLIEiJ_G0000012 | ENSEMBL | Muscar |  |  |  |
| Shrew mouse | *Mus_pahari* | MGP_PahariEiJ_G0000005 | ENSEMBL | Muspah |  |  |  |
| Steppe mouse | *Mus_spicilegus* | ENSMSIG00000018565 | ENSEMBL | Musspi |  |  |  |
| Western wild mouse | *Mus_ spretus* | ENSMSPG00010029592 | ENSEMBL | Musspr |  |  |  |
| Naked mole-rat female | *Heterocephalus_glaber* | ENSHGLG00000035237 | ENSEMBL | Hetgla |  |  |  |
| Northern American deer mouse | *Peromyscus_maniculatus* | ENSPEMG00000030073 | ENSEMBL | Perman |  |  |  |
| Norway rat | *Rattus_norvegicus* | M14434 | NCBI | Ratnor |  |  |  |
| **Carnivors** | | | |  |  |  |  |
| Giant panda | *Ailuropoda_melanoleuca* | OP161053 | NCBI | Ailmel |  |  |  |
| American black bear | *Ursus_americanus* | ENSUAMG00000021733 | ENSEMBL | Ursame |  |  |  |
| Harbor seal | *Phoca_vitulina* | XM_032410496 | NCBI | Phovit |  |  |  |
| Dog | *Canis_lupus familiaris* | ENSCAFG00845012811 | ENSEMBL | Canlupfam |  |  |  |
| Dingo | Canis_lupus dingo | ENSCAFG00020016883 | ENSEMBL | Canlupdin |  |  |  |
| Red fox | *Vulpes_Vulpes* | ENSVVUG00000028725 | ENSEMBL | Vulvul |  |  |  |
| European polecat | *Mustela_putorius* | MT330237 | NCBI | Musput |  |  |  |
| American mink | *Neogale vison* | L07787 | NCBI | Neovis |  |  |  |
| California sea lion | *Zalophus_californianus* | XM_027621286 | NCBI | Zalcal |  |  |  |
| Sea otter | *Enhydra_lutris* | XM_022504288 | NCBI | Enhlut |  |  |  |
| Suricate | *Suricata_suricatta* | XM_029937767 | NCBI | Sursur |  |  |  |
| Cougar | *Puma_concolor* | XM_025927097 | NCBI | Pumcon |  |  |  |
| Cat | *Felis_catus* | KY795140 | NCBI | Felcat |  |  |  |
| Grey seal | *Halichoerus grypus* | XM_036091611 | NCBI | Halgry |  |  |  |
| Weddell seal | *Leptonychotes weddellii* | XM_031018812 | NCBI | Lepwed |  |  |  |
| Northern fur seal | *Callorhinus_ursinus* | XM_073897430 | NCBI | Calurs |  |  |  |
| **Perissodactyla** | | | |  |  |  |  |
| Horse | *Equus caballus* | X75612 | NCBI | Equcab |  |  |  |
| Plains Zebra | *Equus quagga* | XR_006888658 | NCBI | Eququa |  |  |  |
| Donkey | *Equus asinus* | ENSEASG00005001833 | ENSEMBL | Equasi |  |  |  |
| South-central black rhinoceros | Diceros bicornis minor | XM_058550720 | NCBI | Dicbicmin |  |  |  |
| **Cetartiodactyla** | | | |  |  |  |  |
| Pig | Sus scrofa | AK391095 | NCBI | Susscr |  |  |  |
| Cow | Bos taurus | BC122795 | NCBI | Bostau |  |  |  |
| Banteng | Bos javanicus | XM_061432594 | NCBI | Bosjav |  |  |  |
| Wild yak | Bos mutus | XM_070380150 | NCBI | Bosmut |  |  |  |
| Siberian musk deer | Moschus moschiferus | ENSMMSG00000000579 | ENSEMBL | Mosmos |  |  |  |
| Reeves's muntjac | Muntiacus reevesi | XM_065927293 | NCBI | Munree |  |  |  |
| Mishmi takin | Budorcas taxicolor | XM_052648250 | NCBI | Budtax |  |  |  |
| Asian water buffalo | Bubalus kerabau | XM_055539109 | NCBI | Bubker |  |  |  |
| Sumatran serow | Capricornis sumatraensis | XM_068984499 | NCBI | Capsum |  |  |  |
| Sheep | Ovis aries | X54110 | NCBI | Oviari |  |  |  |
| Goat | Capra hircus | JO556991 | NCBI | Caphir |  |  |  |
| Camel | Camelus dromedarius | HE653115 | NCBI | Camdro |  |  |  |
| Llhama | Vicugna pacos | XM_072951498 | NCBI | Vicpac |  |  |  |
| sperm whale | Physeter macrocephalus | XM_024129969 | NCBI | Phymac |  |  |  |
| Sperm whale | Physeter catodon | ENSPCTG00005006801 | ENSEMBL | Phycat |  |  |  |
| Narwhal | Monodon monoceros | ENSMMNG00015020679 | ENSEMBL | Monmon |  |  |  |
| Common minke whale | Balaenoptera acutorostrata | XM_057558766 | NCBI | Balacu |  |  |  |
| North Atlantic right whale | Eubalaena glacialis | XM_061211235 | NCBI | Eubgla |  |  |  |
| Rice's whale | Balaenoptera ricei | XM_059943241 | NCBI | Balric |  |  |  |
| false killer whale | Pseudorca crassidens | XM_067704425 | NCBI | Psecra |  |  |  |
| Common bottlenose dolphin | Tursiops truncatus | XM_073791748 | NCBI | Turtru |  |  |  |
| Common dolphin | Delphinus delphis | XM_069541266 | NCBI | Deldel |  |  |  |
| Beluga whale | Delphinapterus leucas | ENSDLEG00000005288 | ENSEMBL | Delleu |  |  |  |
| Gray whale | Eschrichtius robustus | XM_068564029 | NCBI | Escrob |  |  |  |
| Blainville's beaked whale | Mesoplodon densirostris | XM_060117212 | NCBI | Mesden |  |  |  |
| Long-finned pilot whale | Globicephala melas | XM_060309630 | NCBI | Glomel |  |  |  |
| Pacific White-sided Dolphin | Sagmatias obliquidens | XM_027129095 | NCBI | Sagobl |  |  |  |
| killer whale | Orcinus orca | XM_033416309 | NCBI | Orcorc |  |  |  |
|  | **Chiroptera** |  |  |  |  |  |  |
| Greater horseshoe bat | Rhinolophus ferrumequinum | ENSRFEG00010008208 | ENSEMBL | Rhifer |  |  |  |
| Chinese rufous horseshoe bat  Natal long-fingered bat | Rhinolophus sinicus  Miniopterus natalensis | XR_002139618  XM_016201853 | NCBI  NCBI | Rhisin  Minnat |  |  |  |
| Large flying fox | Pteropus vampyrus | XM_023520504 | NCBI | Ptevam |  |  |  |
| Indian flying fox  Egyptian fruit bat | Pteropus giganteus  Rousettus aegyptiacus | XM_039852393  XM_036224274 | NCBI  NCBI | Ptegig  Rouaeg |  |  |  |
| Greater mouse-eared bat | Myotis myotis | XM_036352654 | NCBI | Myomyo |  |  |  |
| Daubenton's bat | Myotis daubentonii | XM_059686047 | NCBI | Myodau |  |  |  |
|  | **Eulipotyphla** |  |  |  |  |  |  |
| Iberian mole  Hedgehog | Talpa occidentalis  Erinaceus europaeus  **Xenarthra** | XM_037527795 ENSEEUG00000005731 | NCBI  ENSEMBL | Talocc  Erieur |  |  |  |
| Southern two-toed sloth | Choloepus didactylus | XM_037807602 | NCBI | Chodid |  |  |  |
| Armadillo | Dasypus novemcinctus | ENSDNOG00000043741 | ENSEMBL | Dasnov |  |  |  |
|  | **Afrosoricida** |  |  |  |  |  |  |
| Lesser hedgehog tenrec | Echinops telfairi | ENSETEG00000004221 | ENSEMBL | Echtel |  |  |  |
| common brushtail possum  Tammar wallaby | **Marsupials**  Trichosurus Vulpecula  Macropus eugenii | AF321285  EF599616 | NCBI  NCBI | Trivul  Maceug |  |  |  |
| Opossum | Monodelphis domestica | ENSMODG00000009815 | ENSEMBL | Mondom |  |  |  |
| Agile gracile opossum | Gracilinanus agilis | XM_044663076 | NCBI | Graagi |  |  |  |
| Common wombat | Vombatus ursinus | ENSVURG00010007921 | ENSEMBL | Vomurs |  |  |  |
| Koala | Phascolarctos cinereus | ENSPCIG00000015006 | ENSEMBL | Phacin |  |  |  |
| Tasmanian devil | Sarcophilus harrisii | ENSSHAG00000029792 | ENSEMBL | Sarhar |  |  |  |
|  | **Monotremata** |  |  |  |  |  |  |
| Platypus | Ornithorhynchus anatinus | AF491639 | NCBI | Ornana |  |  |  |
| Echidna | Tachyglossus aculeatus | XM_038753111 | NCBI | Tacacu |  |  |  |
|  |  |  |  |  |  |  |  |
